# A laboratory-based Laue X-ray diffraction system for enhanced imaging range and surface grain mapping

**DOI:** 10.1107/S1600576715009097

**Published:** 2015-06-27

**Authors:** William Whitley, Chris Stock, Andrew D. Huxley

**Affiliations:** a Centre for Science at Extreme Conditions, Erskinne Williamson Building, Mayfield Road, Edinburgh EH9 3JZ, Scotland

**Keywords:** laboratory-based Laue X-ray diffraction, grain mapping

## Abstract

Although CCD X-ray detectors can be faster to use, their large-area versions can be much more expensive than similarly sized photographic plate detectors. When indexing X-ray diffraction patterns, large-area detectors can prove very advantageous as they provide more spots, which makes fitting an orientation easier. On the other hand, when looking for single crystals in a polycrystalline sample, the speed of CCD detectors is more useful. A new setup is described here which overcomes some of the limitations of limited-range CCD detectors to make them more useful for indexing, whilst at the same time making it much quicker to find single crystals within a larger polycrystalline structure. This was done by combining a CCD detector with a six-axis goniometer, allowing the compilation of images from different angles into a wide-angled image. Automated scans along the sample were coupled with image processing techniques to produce grain maps, which can then be used to provide a strategy to extract single crystals from a polycrystal.

## Abstract 2

Apparatus is described for the purpose of automated grain mapping of polycrystalline samples within a laboratory environment, using a motorized sample stage and a CCD detector. The limitations that come from using a faster detector of relatively small surface are countered using an automated translation stage.

## 1. Introduction   

White-beam (Laue) X-ray backscatter diffraction is a standard laboratory technique for determining the orientation of single crystals, but it also finds use when extracting a single-crystal from a larger polycrystalline structure. This would be done by taking images with the beam intercepting the sample at various different points and then comparing the patterns seen to determine if a grain is continuous between these points. Although this is a simple laboratory technique, more sophisticated implementation of this method has been used with synchrotron radiation to produce three-dimensional maps of the structure of polycrystals (Chung & Ice, 1999 ▸; Ice & Pang, 2009 ▸). This involves automated capture and indexing of Laue patterns across a sample, which requires a large CCD detector that can obtain a sufficient number of spots for indexing. With modern optics, it is possible to focus the incident beam and extract information on sub-micrometre-sized structures using this approach (Eastman *et al.*, 2002 ▸; Xu *et al.*, 2001 ▸).

In the laboratory environment, for indexing and aligning single crystals, it is usually cheaper to use a plate detector than such a large CCD detector. Furthermore, when separating a large single-crystal from a polycrystalline structure, usually all that is important is knowing where the boundary is of the largest grain, and it is unnecessary to index the whole structure. However, scanning a large sample grown by, for example, Czochralski pulling would be much faster done point-by-point using a CCD detector than with a plate detector. In addition, when scanning such samples (which can often have uneven surfaces and contain heavy atoms) it is much harder to get information on the depth of grains, even with high-energy radiation sources.

The University of Edinburgh employs both plate and CCD detectors for different applications. One of the Laue backscatter setups makes use of photostimulable plates and a scanner, both of which have been recently updated to use a new Fujifilm FCR Capsula XL II system. The Fujifilm system is aimed at medical applications, but was adapted to Laue backscatter imaging by drilling a hole in the centre of the image plates, to allow the beam collimator to pass through. Although this adds the risk of contamination from outside light sources leaking in, this was not found to have any significant impact on the obtained images.

CCD detectors are not produced with holes, meaning that it is not simple to replace such a plate detector with a CCD detector. Photonic Science Laboratories produce detectors in which the backscattered rays either side of the source beam are reflected onto two separate detectors, and the resulting images are stitched together to produce an image with larger area. However, with the extra processing required for each image, the capture cannot be continuous. These considerations led us to decide to use a CCD detector positioned at an angle in a different setup.

A large six-axis automated goniometer has been combined with a CCD camera X-ray detector, which allows users to scan the surface of samples and find the large single grains within a sample using a quick and fully automated procedure. It is also possible to use the goniometer to automatically enlarge the effective area of the detector by rotating and stitching together images that cover different ranges in *k* space.

The equipment used a 2 kW tungsten X-ray tube. The beam produced by this was focused using an optic from XOS, with a focal distance of 140 mm. This had a focal spot size of <0.34 mm, and an intensity gain of >10, when tested at 17.4 keV. This was partly done to increase the beam intensity but also to reduce the size and therefore maximize the resolution of surface scans. The effects of this optic were studied as part of the system calibration.

The CCD detector was supplied by Photonic Science Laboratories and has a sensitive surface area of diameter 66 mm. The detector uses a ‘circle in a square’ configuration, resulting in a dark area (a ‘vignette’) encircling the illuminated region. The goniometer was supplied by Huber (who also made the controller with which the main software controlling the setup interfaces). The goniometer has three rotational axes, as well as three large perpendicular ranges (25, 25, 100 mm) of translational motion.

## 2. Experimental setup   

### 2.1. System calibration   

**Figure 1:**
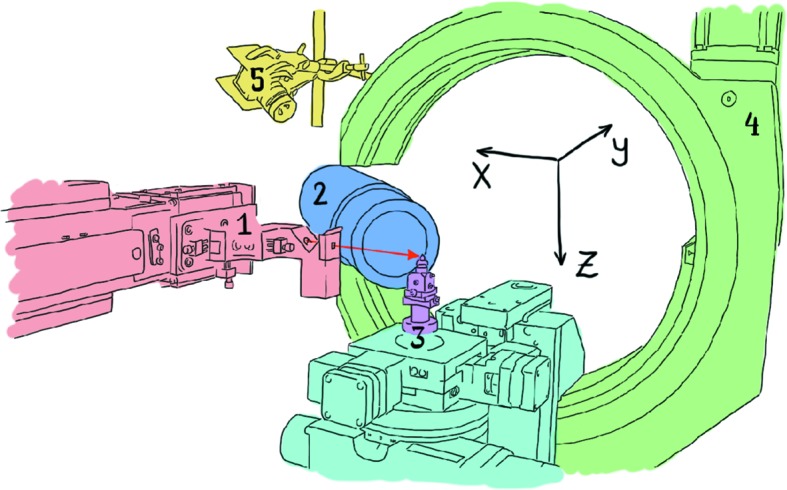
Labelled sketch of the setup. Shown items: (1) X-ray source and focusing optic; (2) detector; (3) sample mounting stage of six-axis goniometer (shown fitted with a smaller goniometer used for system alignment); (4) six-axis goniometer; (5) over-beam webcam. The red arrow shows the path of the incident beam, parallel to the *x* axis.

**Figure 2:**
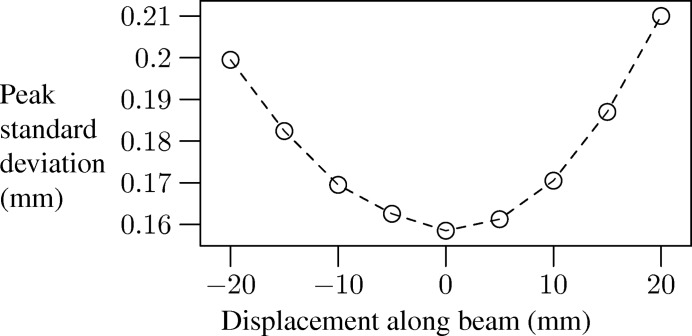
Focusing effect of the XOS optic, showing the reduction of spot size at the centre of the goniometer rotation.

At zero rotation, the *x* axis was calibrated to be co-aligned with the X-ray beam (Fig. 1 ▸). The goniometer sits on air pads which allow us to move it along the *x*- and *y*-axis directions in a controlled manner to achieve this. The alignment was done by traversing a fine needle in the path of the beam and marking the peak in intensity at various positions. By traversing a 100 µm wire across the beam at various distances along it, we were able to examine the focusing behaviour of the optic. Fig. 2 ▸ shows the effects of the beam focusing along the length of the *x* axis.

The detector axis was fixed to intercept the beamline at the centre of rotation, as seen in Fig. 1 ▸. The distance between the centre of the detector and the centre of rotation (*D*) needed to be determined as it is an important variable in most geometry calculations. This was done in two ways. In the first method, the Bragg spot positions were tracked as a sample at the centre of rotation was rotated by an angle ϕ. In the second method, the spot positions were tracked as the sample was translated along the *x* axis. The dependence of the spot positions on these variables was fitted with *D* as a fitting parameter. The first method requires the diffraction point to be at the centre of rotation and is more difficult to fit when the diffraction is not in the plane, but was included for comparison. Both methods gave consistent results and found that the point of detection was at the very front of the detector.

### 2.2. Image capture   

**Figure 3:**
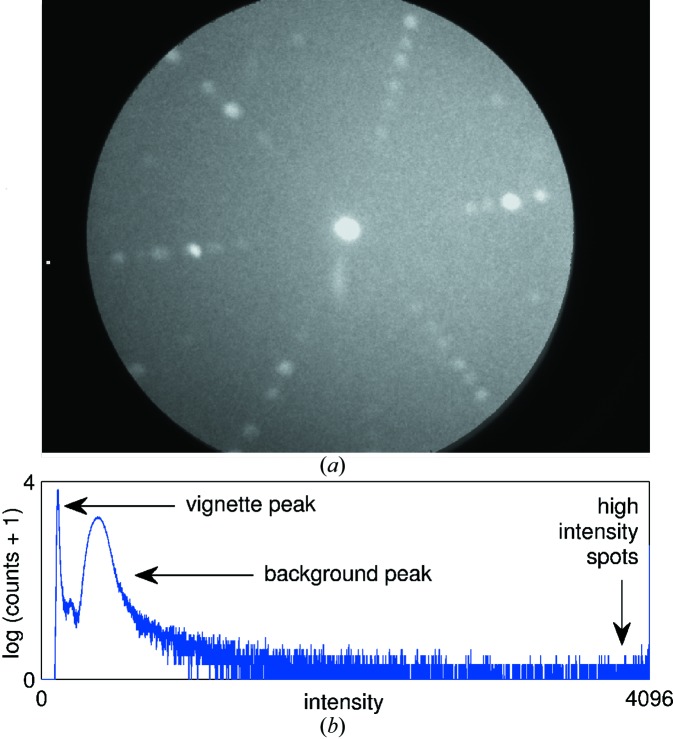
(*a*) An example image produced by the Gemstar detector (this image is an averaged image composed from 3 s-long exposures taken on a sample of MgV_2_O_4_); (*b*) a histogram that shows the different features seen in a typical image.

The X-ray detector allows capture on microsecond timescales, but in reality captures become acceptable after approximately 1 s. It is possible to average many images in order to significantly reduce the signal-to-noise ratio. This system used the *IMAQ Vision* package from National Instruments (Relf, 2004 ▸) for such image processing. Fig. 3 ▸ shows an example averaged image from the detector.

**Figure 4:**
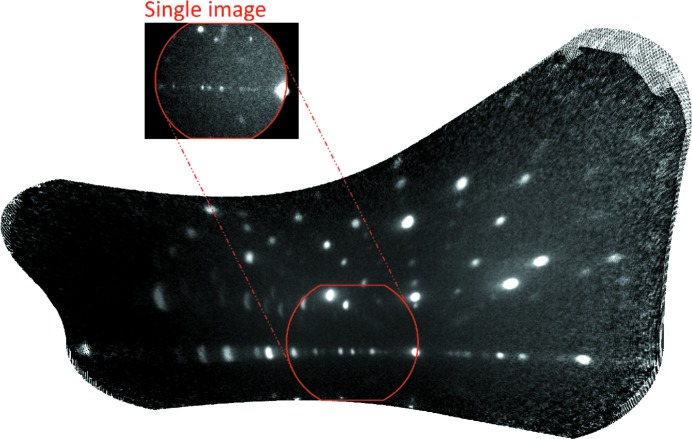
An example image produced by combining images taken at different rotational positions. For this image, the angular range was +20 to −20° around the *z* axis and 0 to 20° perpendicular to this. The red border shows the size of a single unmapped image for comparison. The individual image captured at this rotation is also shown.

A wide-angle capture technique was implemented, in which images taken at different sample rotations were remapped and stitched onto each other in a fully automatic fashion. The remapping process involved calculating the corresponding **k**-vector direction of each pixel in each image, then calculating the rotation necessary to bring this into a reference frame common to all of the images, and finally calculating the diffraction angles for those pixels in this frame. The sharp spike in the image histogram (an example of which is shown in Fig. 3 ▸) at low intensity makes it easy to apply a threshold to remove the counts detected in the image vignette, which would cause distortion to the overlaid images otherwise. Fig. 4 ▸ shows an example of such wide-angle imaging applied to a single-crystal sample of URhGe.

### 2.3. Surface scans   

Surface scans detect that the sample is in the beam by comparing the peak counts with the intensity of the background peak in image histograms. This was more reliable than using the integrated intensity, which can vary significantly across the sample and also depends on the power of the X-rays used. The path finding algorithm looks only for two sample edges or one edge and one limit of motion reached to determine if the sample exists in the current plane of motion. The algorithm works for two-dimensional surface scans at different rotational angles.

### 2.4. Grain mapping   

Determination of grain maps from surface scans first required image processing of all acquired images to automatically find the spot positions within them. This was done by thresholding images at different intensities within a range to the right of the background peak in the image histogram (see Fig. 3 ▸), and also at the saturation value. *IMAQ* particle finding programs (Relf, 2004 ▸) were then used to identify and filter by size the spots seen at each threshold.

After the spots had been found for all images within a sample surface scan, the set of all spots seen could be reduced so that the same spot appearing in multiple images was only included once. To do this, neighbouring images were compared and a limit on spot drift was then necessary as a parameter of the program.

Finally, the characteristic patterns which identified individual grains were determined. Two different approaches were used for this. In the first method, a database of characteristic patterns was built up by a trial and refinement process, using the spot patterns seen in captured images. The database was added to whenever an image displayed a pattern that could not be matched to any already listed. This method was found to be unreliable when automated for several reasons and so an alternative was developed.

The second method looked for Bragg spots which were found to exist within a large region of the sample being studied. Then, the algorithm looked for any significant overlap of such regions. Where these co-existence regions were large, the images within them were assessed to find the most frequently occurring spots, and these were used to form the characteristic patterns. This method has proved more reliable for automatically finding large grains within a polycrystal.

**Figure 5:**
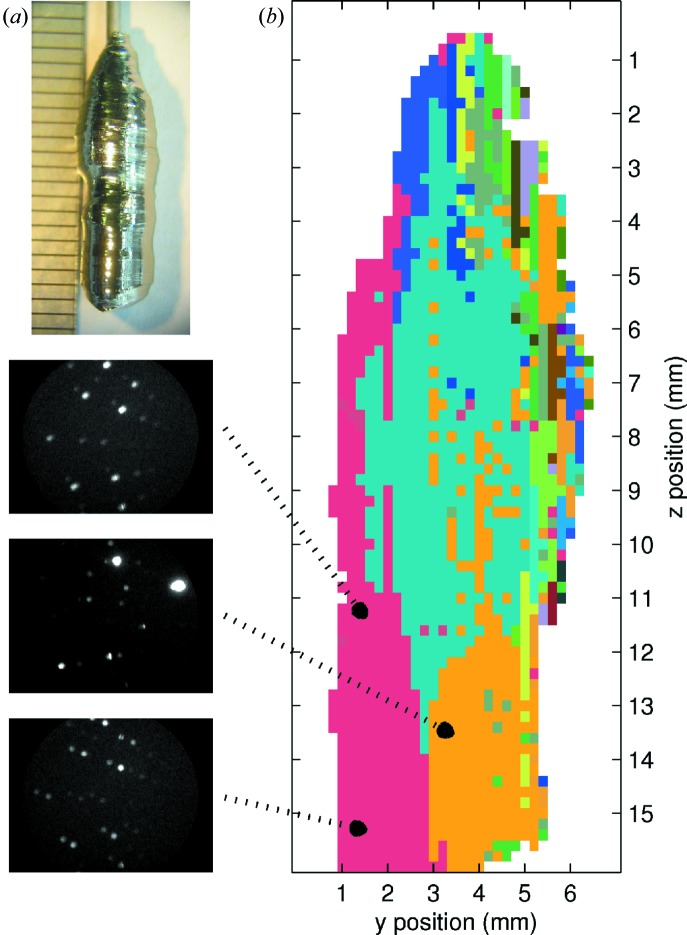
Grain mapping, using the co-existence approach described in the text, applied to a Czochralski-grown sample of UAu_2_. (*a*) Photograph of the sample used. (*b*) Colour-coded grain map. Different colours correspond to different detected patterns within the sample and hence different grains. The images on the left show example patterns which were used for the grain-mapping method.

Once patterns were formed, all images in the scan set were compared with the set of patterns to determine which they fitted the best. An example map produced using this method can be seen in Fig. 5 ▸(*b*), which shows examples of the images that were compared. For this map, a single crystal of UAu_2_ was used, grown by the Czochralski method by J. Schmehr and shown in Fig. 5 ▸(*a*).

## 3. Conclusions   

An X-ray setup has been designed which combines a fast CCD detector with a computer-controlled goniometer to overcome some of the detector limits and also enable automated surface grain mapping. Wide-angled images have been produced, using a pixel-remapping approach. Two approaches to producing automated surface grain maps have been studied, and an approach in which large regions of co-existence between Bragg spots are identified has been found to be the more effective.
